# Commentary: Physical time within human time

**DOI:** 10.3389/fpsyg.2023.1084495

**Published:** 2023-08-14

**Authors:** Yuri Balashov

**Affiliations:** Department of Philosophy, University of Georgia, Athens, GA, United States

**Keywords:** present, temporal experience, persistence, endurance, perdurance, exdurance, stage theory, fission

## Introduction

Gruber et al. (2022) and Buonomano and Rovelli (2023) contribute complementary perspectives to the flourishing debate about the experience of time, currently conducted at the interface of physics, philosophy, psychology, neuroscience, decision theory, linguistics, and other areas. The goal was to connect three vertices of a challenging triangle: the manifest image of time as reflected in common experience, the neuroscientific image of time, and the physical concept of time, which was initially hostile to both. Reconciliation is sorely needed but difficult to achieve.

Part of the problem lies in the substantive disagreement about what temporal experience involves in the first place (Skow, 2015; Prosser, 2016; Callender, 2017; Phillips, 2017; Sullivan, 2018; Sattig, 2019; Miller and Wang, 2022). At some approximation, which appears to be adopted by Gruber et al. and Buonomano and Rovelli, there are three core aspects to our manifest image of time: (i) the notion of a unique objective present (the “time of our lives”), (ii) the perception of time flow, and (iii) an asymmetry between the past and future directions of time: We think of the past as fixed and of the future as open, and we have memories of the former but not of the latter. All of that is difficult to square with the physics of time, which, in Callender's words, “suggests that manifest time is more or less rubbish” (Callender, 2017, p. 2). Quite apart from that, the notions of “experiencing the present” and “time flow” have proven to be singularly elusive and resistant to precise definition, which, of course, makes the problem philosophically interesting.

In the following section, I have attempted to focus on a particular aspect of the experience of the present which, in my view, has received insufficient attention.

## Time and persistence

Gruber et al. (2022) and Buonomano and Rovelli (2023) (and many other participants in the debate) share the view known as the “Block Universe,” according to which different times and their contents are similar to different places and their contents—all equally real; indeed, one way to think of times is to identify them with special regions of spacetime (e.g., achronal Cauchy surfaces), but many of those who subscribe to this view tend to believe that objects persist over time by *enduring*—by being “wholly present” (or “multilocated”)—at many instantaneous spacetime regions. Denying this latter claim does not amount to denying *persistence* altogether [here, I disagree with Gruber et al. (2022) and side with Miller and Wang (2022)]. It does add more to the analogy between time and space: Objects may persist through time much like rivers persist through space, by having distinct parts at different times. This mode of persistence, known as perdurance, is favored by some philosophers (e.g., Lewis, 1986; Heller, 1990) and, according to recent empirical research (Baron et al., 2022), may not be so foreign to common sense as previously believed. But there is a third view of persistence, known as stage theory, on which, rather than having distinct temporal parts or stages at different times, ordinary objects are stages (Hawley, 2001; Sider, 2001). They can still be said to persist by *exduring*—by having temporal *counterparts* at other times—by analogy with modal counterparts inhabiting disconnected regions of the Lewisian “pluriverse” (Lewis, 1986). This official statement of stage theory is also Block Universe-friendly but may be much less intuitive. The best arguments in its favor involve rather abstract philosophical conundrums of material coincidence and vagueness (Sider, 2001), but I contend that it can also be supported by reflection on a central feature of our temporal experience (Hoy, 1978; Torre, 2010; Parsons, 2015; Skow, 2015; Balashov, 2017), especially when this is followed by a leap of imagination inspired by influential thought experiments (Parfit, 1971, 1984, 2008).

## Time and fission

In his groundbreaking work, Parfit (1971, 1984, 2008) invites the readers to join him in exploring the moral and metaphysical implications of a *fission* scenario in which a person, Ed, is physically and/or psychologically continuous with two future persons, Ted and Fred. Assuming the process goes smoothly (imagine Ed performing a mental operation of adding 47 and 38 just before the fission, and Ted and Fred both saying “85” immediately thereafter), we can suppose Ted to be happy and Fred to be sad (any pair of incompatible mental states will do). Suppose further that Ted says he is happy, and Fred says that he is sad. Each of them is unaware of what the other is feeling and saying. Putting ourselves in Ed's shoes, can we say that he *will be* happy or sad? More fundamentally, can we say of Ed that he is identical (across time) with Ted, Fred, or both? We can assume that Ed's relations to Ted and to Fred have “all the matters” for survival (i.e., physical and/or psychological continuity) and are, in that respect, on a par. This suggests that if Ed is identical with Ted, he is also identical with Fred, but one entity cannot be identical with two. The only alternative is to say that Ed is identical with neither of them. Much of Parfit's work can be read as denying a substantive difference between these two alternatives. If Ed's relation to both future persons has everything that matters, it is as good as it can get and may be sufficient for survival.

While Parfit's focus was on the philosophical implications of fission, he was aware that his scenario involves not only the *personal* and *spatial* dimensions of “self-location” (Ed may be wondering *who he is* after fission, Ted or Fred; relatedly, he may be wondering *where he is*) but also a temporal dimension (Ed may be wondering *when it is*). This becomes clear from Parfit's extended discussion of our attitudes toward future persons—ourselves as well as our relatives and friends, with no clear boundaries between them. This leads Parfit to the metaphysics of the self “scattered” or “fragmented” across all three dimensions: spatial, personal, and temporal, which, in turn, may have a distant similarity with Gruber et al.'s notion of the “impermanent” or “ephemeral” self (Gruber et al., 2022, p. 4f). I submit that it also offers a useful perspective on the stage theory of persistence: Just as Ed may be “split” between Ted and Fred (and their two spatial locations), he may be “split” among multiple temporal locations hosting his numerically distinct stages.

## Time and self

**Figure 1:**
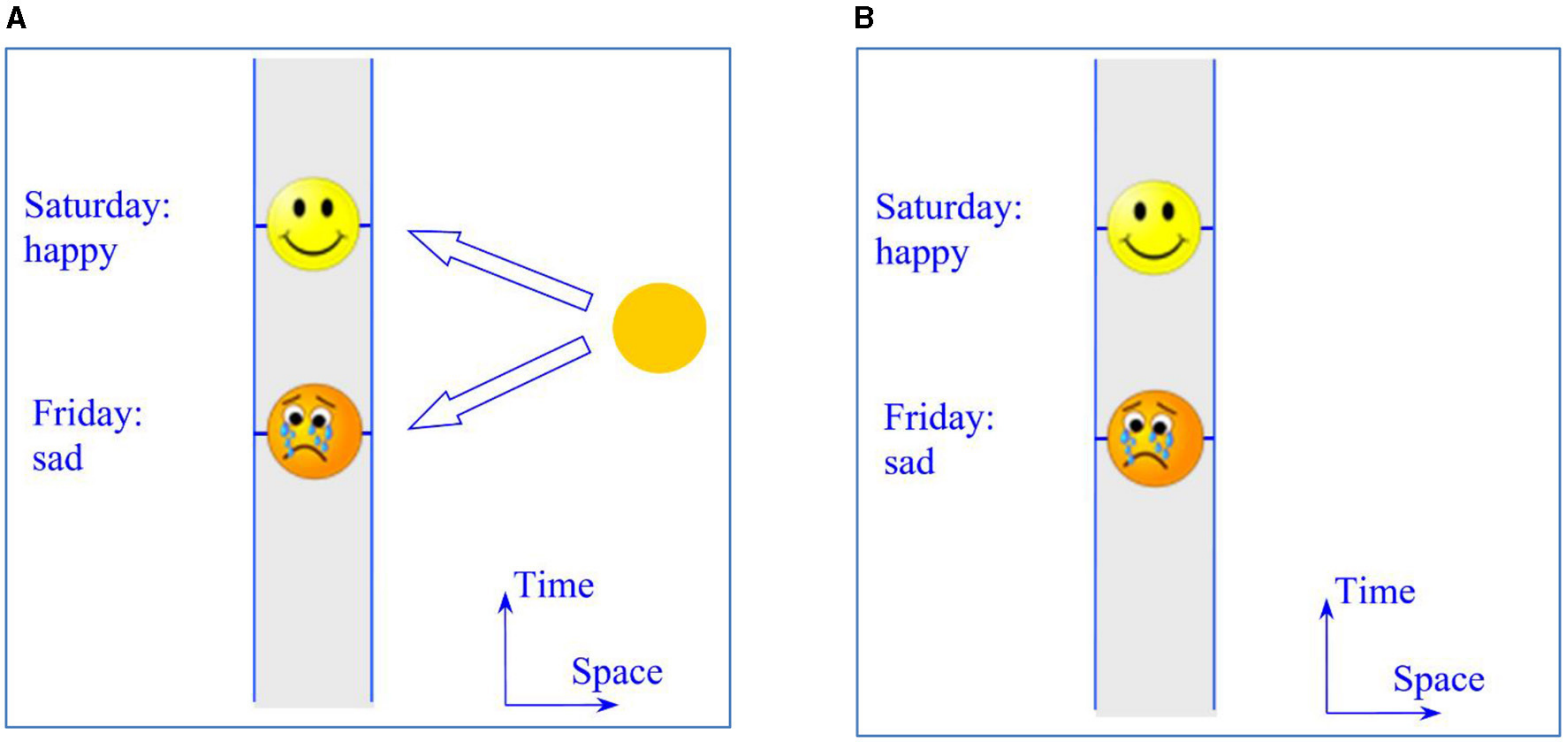
Ed's feelings in the Block Universe **(A)** with endurance and **(B)** with exdurance. The shaded regions represent Ed's path in spacetime.

Suppose Ed is sad on Friday and happy on Saturday and put yourself in his shoes. Next, situate this scenario in a Block Universe with *endurance*. This, I think, raises the problem of explaining Ed's *present experiences* and his beliefs about them: He is sad (let us assume) and believes it is Friday. The bottom arrow in Figure 1A represents his perspective on the Block Universe, tainted with sadness, but there are many other perspectives, including the happy one (the top arrow in Figure 1A, modeled after Figure 2 in Balashov, 2017), and nothing in the Block Universe favors one of them over another. What then explains Ed's belief that he is viewing the Block Universe exclusively from the Friday perspective filled with sadness, *rather than* exclusively from the Saturday perspective filled with happiness and joy? If Ed *endures*—if he is wholly present on Friday as well as Saturday—then nothing in the Block Universe allows his different temporal experiences to be “compartmentalized” the way they seem to be. To adapt Callender's (2017) term (he will disapprove this usage of it), the “ontic voltage” of the present experiences is too high for anyone to *endure*.

Suppose, in contrast, that Ed *exdures*—that, instead of the selfsame enduring Ed, there are multiple stages of him, each representing his perspective on the single Block Universe (Figure 1B, modeled after Figure 3 in Balashov, 2017). His Friday stage is sad and finds itself exclusively on Friday, thus giving Ed an illusory belief in the exclusive presence on Friday and his exclusive sadness, but the same can be said of his Saturday stage and the corresponding illusory belief that goes along with it. Importantly, in having the Friday belief and the corresponding experiences, Ed is *not aware* of having the Saturday belief and its attendant experiences. This is parallel to Parfit's reasoning about fission and its consequences. If the self is “scattered” across times in the same way it is scattered across places and persons, then the problem of the present experiences and the problem of the “split self” are resolved in the same way. As already noted, Parfit's work outlines the general shape of such a unified explanation; but it could, and should, be made more explicit.

This opening move is open to many objections, including the tendency to dismiss it as based on an obvious confusion between the tensed and tenseless uses of “view” and “feel,” insufficient attention to the indexical nature of the phenomena described in this scenario, and more. I believe these objections can be addressed by further developing the scenario (Balashov, 2017). The problem of explaining the nature of the present experiences arises quite early in the process of reconciling the manifest image of time with its scientific image, and it appears to be relatively independent of the issues of time flow and time direction. It may be related to what Buonomano and Rovelli call “the special role of the present” and Gruber et al. discuss under the heading of “no unique present.” In any case, the problem keeps coming back in various guises (Hoy, 1978; Parsons, 2015; Skow, 2015), which, I think, calls for more attention to it.

## Author contributions

The author confirms being the sole contributor of this work and has approved it for publication.
